# Noise-robust recognition of wide-field motion direction and the underlying neural mechanisms in *Drosophila melanogaster*

**DOI:** 10.1038/srep10253

**Published:** 2015-05-14

**Authors:** Yoshinori Suzuki, Hideaki Ikeda, Takuya Miyamoto, Hiroyoshi Miyakawa, Yoichi Seki, Toru Aonishi, Takako Morimoto

**Affiliations:** 1 Interdisciplinary Graduate School of Science and Engineering; Tokyo Institute of Technology; Yokohama, Kanagawa, 226-8502, Japan; 2 Japan Society for the Promotion of Science, Tokyo, Japan; 3 School of Life Sciences; Tokyo University of Pharmacy and Life Sciences, Hachio-ji, Tokyo, 192-0392, Japan

## Abstract

Appropriate and robust behavioral control in a noisy environment is important for the survival of most organisms. Understanding such robust behavioral control has been an attractive subject in neuroscience research. Here, we investigated the processing of wide-field motion with random dot noise at both the behavioral and neuronal level in *Drosophila melanogaster*. We measured the head yaw optomotor response (OMR) and the activity of motion-sensitive neurons, horizontal system (HS) cells, with *in vivo* whole-cell patch clamp recordings at various levels of noise intensity. We found that flies had a robust sensation of motion direction under noisy conditions, while membrane potential changes of HS cells were not correlated with behavioral responses. By applying signal classification theory to the distributions of HS cell responses, however, we found that motion direction under noise can be clearly discriminated by HS cells, and that this discrimination performance was quantitatively similar to that of OMR. Furthermore, we successfully reproduced HS cell activity in response to noisy motion stimuli with a local motion detector model including a spatial filter and threshold function. This study provides evidence for the physiological basis of noise-robust behavior in a tiny insect brain.

Animals respond to various kinds of sensory stimuli presented in the environment. In laboratory conditions, one can design a simple and ideal sensory stimulus to investigate the neural mechanisms of sensory information processing. However, in actual native habitats, animals rarely receive simple stimuli because the sensory inputs change spatiotemporally and kaleidoscopically and such changes often appear simultaneously, making the signal noisy. Animals have to respond appropriately and robustly even to such vague stimuli. One of the most important issues in neuroscience is how the brain processes ambiguous information to guarantee robust behavioral reactions. Answering this question will provide a better understanding of the neural mechanisms controlling behavior in natural environments and may further contribute to the development of robust engineering operating systems.

The optomotor response (OMR) is an important behavioral reaction to a sensory stimulus; it is observed in most sight-reliant animals, from vertebrates to invertebrates. The OMR is a compensatory reaction to wide-field motion or ego-motion to stabilize the retinal image and is thought to be important for course control during flight^3^, walking^5^, swimming^6^, and escaping^7^. A considerable number of studies have been conducted on the neural and computational mechanisms underlying the OMR in the blowfly visual system^8^ and more recently in *Drosophila melanogaster*^9^. Both systems possess almost the same neural structures and functions^0^. Retinotopically processed motion information converges in a set of wide-field motion-sensitive neurons called lobula plate tangential cells (LPTCs)^1^. LPTCs involve three horizontal system (HS) cells that respond to horizontal wide-field motion stimuli^2^. During preferred direction (PD) motion stimulation, HS cell membrane potentials are depolarized, whereas they are hyperpolarized during anti-preferred or null direction (ND). Previous studies have shown that activation of HS cells induces the OMR^4^ and further reported that defects in LPTCs reduce walking OMR^5^, suggesting a strong association between HS cell activity and the OMR. Response properties of either HS cells or the OMR in relation to several stimulus features such as speed, contrast, and pattern of motion stimulus have been thoroughly investigated. However, few studies have simultaneously investigated both the OMR and HS cell activity in response to motion stimuli with added noise, and the relationship between the OMR and HS activity in this context remains poorly understood.

Here, we studied the properties of the OMR in response to wide-field motion stimuli with random dot noise and compared it with the neural activity of HS cells. Our results obtained with signal classification theory, receiver operating characteristic (ROC) analysis, revealed that flies robustly process wide-field motion direction even if the stimulus contains a considerable amount of noise, and that the discriminative performance of HS cell activity to motion directions revealed by ROC analysis accounts for this noise-robust sensation. Direct comparisons between behavior and neural activity by ROC analysis revealed the quantitative correspondence of robust performance under noisy conditions. Furthermore, we performed simulation studies to examine the possible neural mechanisms underlying this feature of HS activity. This study provides a physiological basis for robust processing of wide-field motion stimuli with noise.

## Results

### Robust processing of wide-field motion with random dot noise

**Figure 1:**
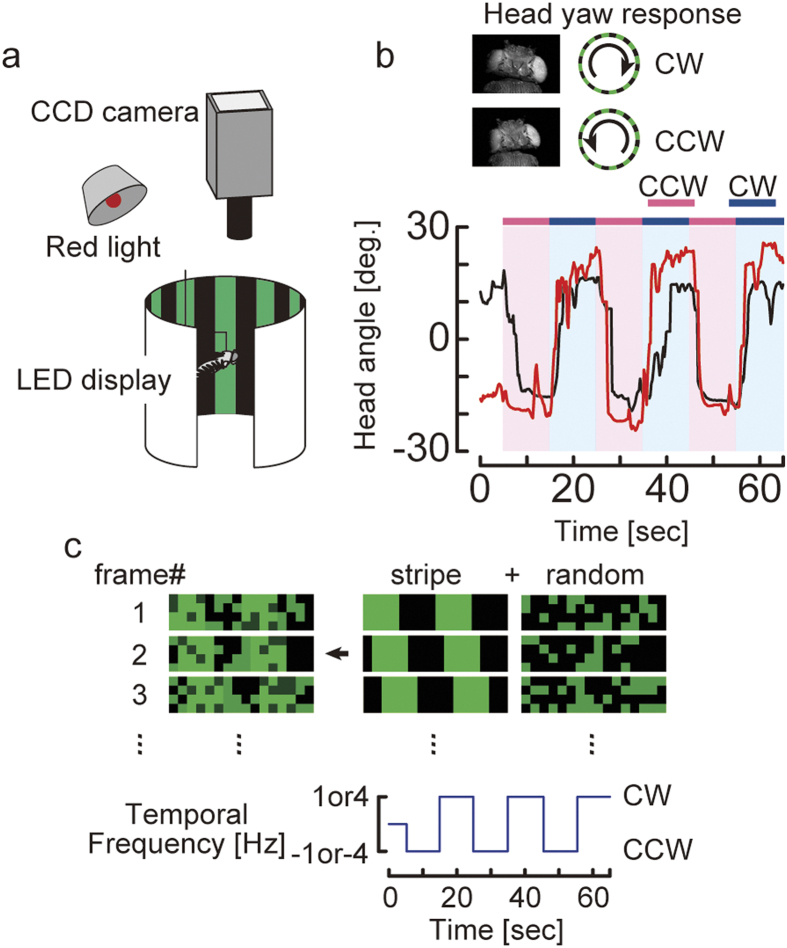
Behavioral experiment in a tethered fly. (**a**) Schematic diagram of the apparatus. A fly is fixed to a steel pin and placed in the center of an LED arena that displays visual stimuli controlled by a PC. Head movements are recorded by a CCD camera above the arena to measure the OMR. (**b**) When presented with a yaw rotation motion stimulus, the fly rotates its head to follow the motion (top). The head yaw angles can be computed from the recorded videos. Examples of two individual traces of the head yaw response during CW and CCW motion (bottom). The original position is not always centered because flies move their heads freely before stimulus presentation. (**c**) Visual stimuli are constructed by the superimposition of random dot noise on a panoramic vertically striped square-wave grating. When a random dot overlaid a bright bar of the stripe pattern, the luminance of the random dot was subtracted from that of the bright bar. Thus, the average LED display intensity was kept nearly constant during motion presentation. The temporal sequence of an individual trial is shown (bottom).

We tested the OMR to motion stimuli with a computer-controlled LED display (Fig. 1a). As shown in Fig. 1b, flies turn their heads toward the direction of motion during stimulus presentation. In order to quantify the response, we measured the head yaw angle, which has been established as an index of the OMR^8^. To test the effects of reduced stimulus reliability on wide-field motion processing, we precisely controlled the signal-to-noise ratio (SNR) of the moving stimulus. Therefore, rotating vertical stripes superimposed with several intensities of random dot noise were used as motion stimuli (see Supplemental videos S1,S2,S3,S4,S5,S6). The pattern turned in clockwise (CW) and counter-clockwise (CCW) directions. We used two different temporal frequencies (1 and 4 Hz, Fig. 1c; see Methods).

**Figure 2:**
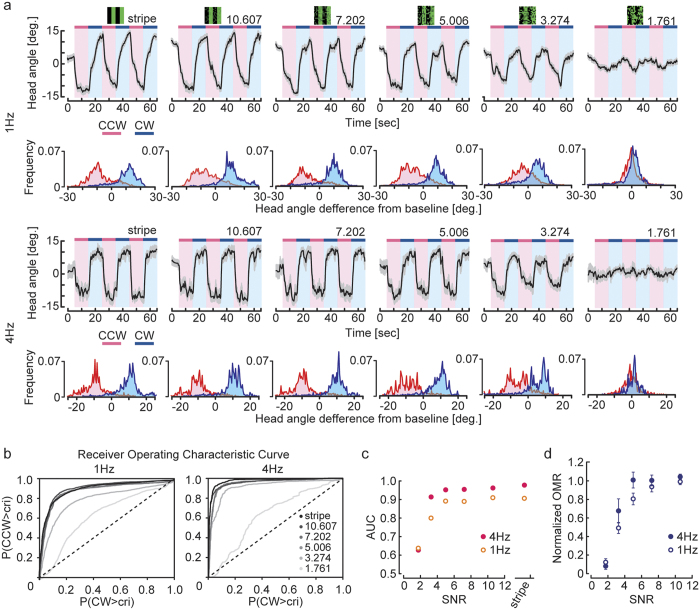
Flies can robustly discriminate wide-field motion directions under noisy conditions. (**a**) Average of head yaw responses ±s.e.m. (upper panels) and distributions of head angle (lower panels) shown over the range of SNR at 1 and 4 Hz temporal frequencies. Stimulus patterns and SNR are depicted above each figure. Stripe denotes the stripe pattern without noise (red, CCW rotation; blue, CW rotation; n = 20 flies, 36 trials at 1 Hz temporal frequency; n = 9 flies, 17 trials at 4 Hz). (**b**) ROCs for the six pairs of CCW-CW response distributions illustrated in a. Increased separation between CCW and CW response distributions leads to an increased upward deflection of the ROC away from the diagonal. (**c**) Area under the ROC curve (AUC) illustrated in b. A ROC curve along the diagonal indicates that the fly cannot distinguish between CCW and CW rotations, and the AUC is 0.5. As the ROC curve approaches the left axis and upper limit, it indicates that the fly reliably distinguishes between CCW and CW, and the AUC is 1.0. (**d**) Mean ±s.e.m. of normalized OMR illustrated in a.

We evaluated head yaw responses over a range of SNRs (Fig. 2). Flies react to motion stimuli even with substantial amounts of added noise (SNR = 3.274) at both temporal frequencies. However, when the SNR of the stimulus was <3.274, flies no longer respond (Fig. 2a). In order to quantify how accurately flies could discriminate between the two directions of moving stimuli, we created distributions of the head yaw response during stimulus presentation (Fig. 2a second and fourth row, see Methods) and applied a ROC analysis to these distributions. The ROC analysis is a classical and commonly used method to evaluate performance of perceptual detection and allowed us to directly and quantitatively compare the discriminative capacity between behavior and neural activity^9^. The distributions of head yaw responses to CW and CCW motion directions were separate until the SNR reached a moderate level (SNR = 5.006) and then moved closer as noise level increased. When the SNR reached the highest noise level (SNR = 1.761), the two distributions were no longer distinguishable (Fig. 2a rightmost panels). Thus, as noise levels increase, the upward deflection of the ROC curves observed until the moderate noise level (SNR = 5.006) gradually decreased and finally approached the diagonal as noise level reached the maximum (Fig. 2b). We subsequently calculated the area under the ROC curve (AUC). The AUC is a good indicator for quantifying the discriminative performance between different motion directions (see Methods). At both temporal frequencies, the AUC value remained close to the value for the stripe pattern (i.e., without noise) until noise reached a critical level, and the AUC value rapidly decreased (Fig. 2c). Overall, discriminative performance seemed to be slightly higher at 4 Hz than at 1 Hz. We suggest that this frequency-dependent difference in AUCs is caused by an underestimation due to the slower response to 1-Hz stimulation, as evident in the first row of Fig. 2a, rather than a difference in sensitivity to noise. In conclusion, these results indicate that flies can correctly discriminate rotating wide-field motion directions even when the SNR of the motion stimulus is quite low.

Next, we examined the effect of reduced stimulus reliability on the head turning angle amplitude of OMR. We calculated the difference of the average head angle between CW and CCW during the last 5 s of stimulus presentation, and normalized it with the OMR during stripe stimulation without noise (normalized OMR). Similar to the AUC, the normalized OMR remained close to the value under stripe pattern motion until the noise reached a critical level, and then decreased rapidly (Fig. 2d). These results indicate that OMR amplitude, as well as the discriminative performance revealed by the AUC, showed non-linear properties to motion directions under varying noise.

### Discriminative capacity of HS cells revealed by ROC analysis is highly correlated with that of OMR

**Figure 3:**
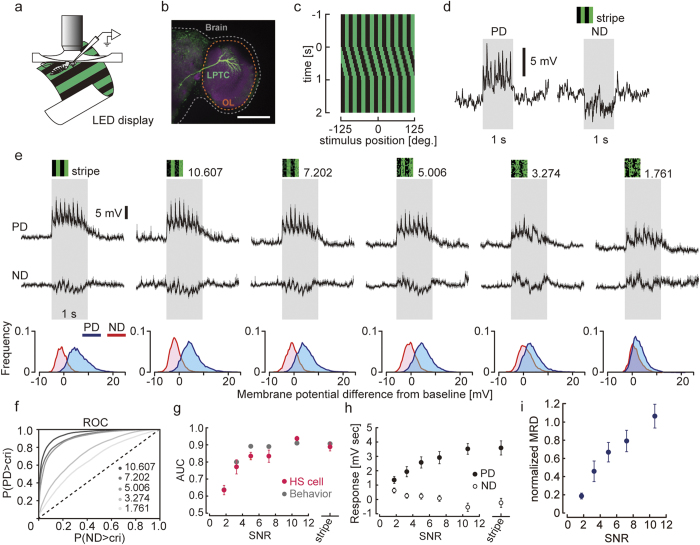
Strong correlation between the discriminative capacity of the OMR and HS cells to motion stimuli with noise. (**a**) Schematic diagram of the recording apparatus. (**b**) A recorded biocytin-filled HS cell (green) located in the right hemisphere (posterior view). Scale bar = 100 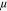
m. OL, optic lobe (**c**) A space-time plot of the rotation stimuli. Each bar is 15^°^ in azimuthal extent, and the temporal frequency is 1 Hz. (**d**) Example of response of a single right HS cell to a stripe pattern moving in PD (CW) or ND (CCW) at a temporal frequency of 1 Hz. The gray-shaded region indicates the period when visual stimuli were in motion (1 s). HS cells are depolarized or hyperpolarized during PD or ND, respectively. (**e**) Average membrane potential ±s.e.m. of an HS cell to visual stimuli of different SNR moving in PD and ND (top panel). The bottom panel shows the distributions of baseline-subtracted membrane potentials during stimulus presentation (red, ND; blue, PD). We noted that HS cells also depolarized during ND motion when the SNR of the stimulus was at a low level. This is speculated to be due to random dot blinking because the random dot pattern becomes a dominant component in the visual stimulus at low SNR levels. (**f**) ROCs for the five pairs of PD-ND response distributions illustrated in e. (**g**) Mean ±s.e.m. of AUC (red, HS cell, n = 10 cells). The equivalent behavioral data from Fig. 2c (at 1 Hz) is added (gray filled circles). (**h**) Mean ± s.e.m. of baseline-subtracted responses to PD and ND directions over a range of SNR. Responses of individual cells were calculated by integrating baseline-subtracted mean membrane potentials over the trials during motion presentation (n = 10 cells). (**i**) Mean ±s.e.m. of normalized response difference between PD and ND illustrated in h (normalized MRD).

To investigate the neural basis underlying the robustness of the OMR, we recorded membrane potentials of HS cells to wide-field motion stimuli with added noise using a whole-cell patch clamp technique (Fig. 3a). As reported previously^9^, we also observed that HS cells depolarized or hyperpolarized during the presentation of ipsilateral front-to-back preferred direction (PD) or back-to-front null direction (ND) horizontal motion, respectively (Fig. 3d). Our PD motion stimuli elicited increases in membrane potential, including spike-like transient depolarization. These spike-like changes in membrane potentials were often elicited by the moving patterns we used (see Methods). As indicated in Fig. 3e, we presented rotational motion with the same range of SNR as in our behavioral experiments described above with 1 Hz temporal frequency. The amplitude of membrane potential changes in response to PD motions decreased with increasing noise level (Fig. 3e). To quantify the discriminative performance of HS cell activities, we analyzed the distributions of HS cell membrane potentials, from which baseline amplitude was subtracted, during stimulus presentation and computed the ROC for each pair of distributions (Fig. 3e bottom panel and Fig. 3f). The upward deflection of ROC curves was kept until a moderate noise level (SNR = 5.006) because there was minimal overlap between the two distributions of membrane potentials in response to PD and ND motion stimuli. As the SNR further decreased, the ROC curves approached the diagonal because the two distributions overlapped almost completely (Fig. 3f). The AUC value remained close to the value in the stripe condition until a moderate noise level and then decreased rapidly (Fig. 3g). This was remarkably similar to the robust non-linear property of the behavioral AUC (Figs. 2c, 3g).

On the other hand, we found that changes in the membrane potential amplitude of HS cells to noisy motion stimuli were proportionally reduced with increasing noise (Figs. 3h, 3i). We analyzed the neural response by integrating the baseline-subtracted mean membrane potential during motion stimulus presentation. The response to PD motion increased and that to ND motion decreased with increasing SNR (Fig. 3h). We calculated the differences between responses to PD and ND stimuli (mean response difference, MRD), and normalized it with the stripe motion stimulus (normalized MRD). This normalized measure linearly decreased with increasing noise levels (Fig. 3i). These results indicate that HS cells robustly discriminate wide-field motion directions even when the stimulus contains a considerable amount of noise; however, the normalized MRD was easily affected by noise. Thus, the behavioral discriminative capacity can be accounted for by the neuronal discriminative capacity revealed by ROC analysis but not by the direct measurement of changes in the membrane potential amplitude.

### An elementary motion detector model with a spatial filter and threshold function reproduces HS cell activity in response to noisy motion stimuli

**Figure 4:**
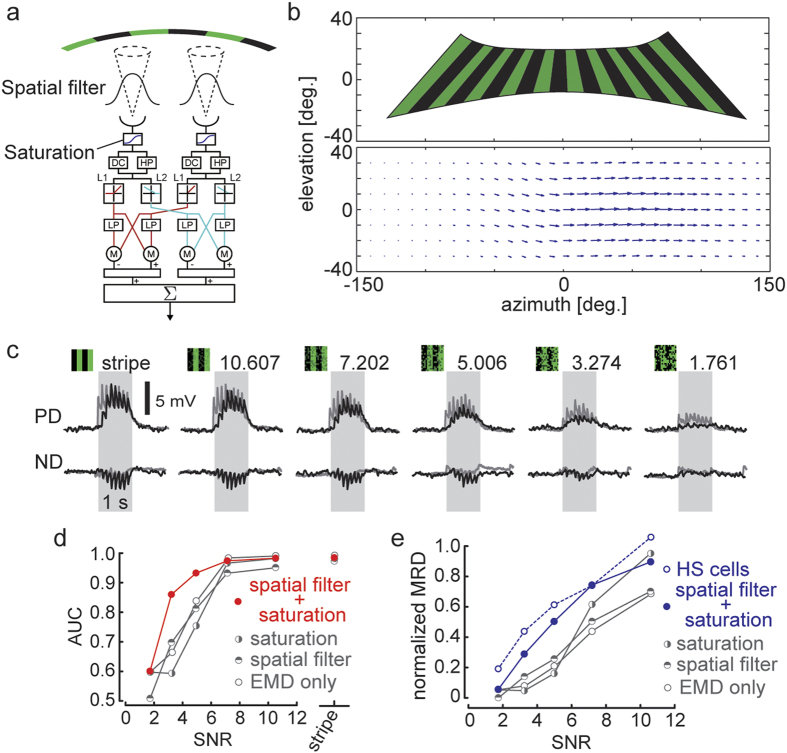
EMD model with added spatial filter and threshold function reproduces the experimental results.(**a**) The structure of our model. First, the visual stimulus is filtered with a 2D Gaussian function (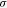 = 7.5°), and the filtered signal is passed through a sigmoid function (threshold function). The sigmoidal output is processed by the 2D array of a 2-Quadrant-Detector model (see Methods). HP, temporal first-order high-pass filter (*τ* = 250 ms); DC, passing 10% of the original signal; LP, temporal first-order low-pass filter (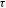 = 150 ms); M, multiplication; Sigma (Σ), nonlinear integration. (**b**) Putative 2D appearance of the LED display from the viewpoint of a tethered fly (top). The spatial distribution of dendritic integration for the model cells (bottom). We constructed the vector field of spatial weight factors 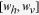 adjusted for the receptive field of the right HS cell^4^. The length and orientation of each vector indicates the level of sensitivity and the preferred direction of local motion detector, respectively. (**c**) Average responses of the model cell (10 trials) to visual stimuli with various SNR in both PD and ND stimulations at a temporal frequency of 1 Hz (black). Average membrane potentials of 10 cells are also presented (gray). The gray-shaded region indicates when visual stimuli were in motion. (**d**) Mean AUC (red, contains both the spatial filter and threshold function as illustrated in a; gray, contains either the spatial filter or threshold function or none of the two). (**e**) Mean normalized MRD (10 trials) for each model (blue, contains both the spatial filter and threshold function [filled circles] and HS cells illustrated in Fig. 3i [open circles]; gray, contains either the spatial filter or threshold function or none of the two).

Next, we focused on the neural architecture and implementation underling HS cell coding. To address this problem, we used a mathematical modeling approach with an elementary motion detector (EMD) model that is thought to reproduce LPTC activities to wide-field motion stimuli^0^. In this study, we added a spatial filter and sigmoidal threshold function, which are generally used to express the spatial receptive field properties and nonlinear response properties of visual neurons to the EMD model to reproduce the experimental results (Fig. 4a). As indicated in Fig. 4c, our model was applied to both PD and ND motion stimuli over the same range of SNR as in our experiments described above. The activities of HS cells observed in our experiments were successfully reproduced by our model simulations. To demonstrate the effectiveness of the spatial filter and threshold function, we compared the model performance with and without these two components. To quantify the discrimination performance of these different cases, we computed the AUC of the model. With both components, the AUC remained close to the value in the stripe condition until the noise reached a critical level and then rapidly decreased as observed in the real HS cells (Fig. 4d). When the SNR was <5.006, the AUC slope of the model with both filters matched the experimental data better than the other models with fewer components. The slope of the discriminative performance is an important index to evaluate robustness to noise. In conclusion, the combination of the two filters was able to improve the performance of the original EMD model and successfully reproduced the experimental results.

Next, to investigate how the modeled MRD was affected by the increase in noise, we normalized MRD with the response to the stripe pattern without noise. As shown in Fig. 4e, the normalized MRD values markedly declined as SNR decreased in the models without any added components and in those with either threshold function or spatial filter. However, the model with both components qualitatively reproduced the normalized MRD values of the experimental result as a function of the SNR. Thus, the model with spatial filter and threshold function accurately reproduced HS cell activities elicited by motion stimuli with added random dot noise.

## Discussion

Robust and reliable behavioral performance in a noisy environment and the underlying neural mechanisms have been attractive subjects for studying the function of the neural system. A number of studies of the visual system have focused on robust information processing by investigating the effects of photon and motion noise^3^. These studies mainly observed the effects of noise on either behavioral aspects or neural activity. Few studies investigated both behavior and the underlying neural activity, although some primate studies reported effects of noise on motion perception and neural activity^4^. However, because of the enormous number of neurons and the complexity of the primate visual system, it might be difficult to uncover the precise response properties of the behavior and its relationship with neural activity in such a large brain.

Here, we investigated both behavior and neural activity in the tiny *Drosophila* brain and their discriminative capacity for panoramic motion direction embedded in random dot noise. We showed the robust ability of flies to discriminate wide-field motion directions under considerable amounts of noise (Fig. 2). Moreover, the discriminative capacity of HS cells for movement direction strongly correlated with behavioral performance of the OMR (Fig. 3). This robust discriminative capacity of HS cells could only be revealed by ROC analysis because the membrane potential changes of the HS cells were proportionally reduced with increasing noise. Our results provide the first evidence that flies with their tiny brains can correctly and robustly react to motion stimuli buried in a noisy environment at both the behavioral and neural level.

Furthermore, we investigated neural architecture and implementation in HS cell coding with a mathematical modeling approach. In general, the local motion detector model is highly influenced by and vulnerable to local noise in motion stimuli because it extracts motion information from temporal patterns of light intensity at adjacent locations within narrow regions. Therefore, the local motion detector model severely underestimates the response to motion stimuli with noise^6^. In order to elucidate which mechanisms facilitate the noise-robust discriminative responses of HS cells, we added a Gaussian spatial filter and threshold function to the local motion detector model. We successfully reproduced the experimental results when we added a spatial filter and threshold function to the EMD model (Fig. 4). These results suggest that spatial filtering and binarization of visual inputs are essential to generate the distinctive features of HS cell activities elicited by motion stimuli with added noise. The spatial filter smoothed the stochastic signal fluctuations with a weighted average and reduced the contrast of visual patterns, while the threshold function emphasized and reconstructed the contrast reduced by spatial filtering. Thus, we hypothesize that smoothing out noise and contrast enhancement, which are accomplished by these two components, improve the SNR of visual inputs, which allows our model to account for the response properties of HS cells to noisy motion stimuli. With regard to the response of LPTCs, Schnell *et al.* (2010)^7^ showed that the response amplitudes of HS cells to PD motion saturated when the visual pattern contrast was high. In this study, we also observed a saturated response to the high SNR stimulus. The saturation property of the threshold function in our model is also required to explain the response properties of HS cell activity.

What are the physiological correlates of these two components in the real fly visual system? Lamina monopolar cells are one candidate for the spatial filter. In *Drosophila*, one lamina cell receives visual channel outputs from six neighboring retina cells^8^ and seems to be involved in spatial summation. It is well known that in the visual system of animals living in dark habitats, several interneurons and ganglion cells, each of which having a wide dendritic field, achieve spatial summation to improve visual performance under dim light conditions^9^. For the threshold function, candidates in the fly visual system would have typical properties of neural cells, such as non-linear neural responses to light intensity and non-linear synaptic transformation^2^. Further anatomical and functional analysis of visual motion networks will shed light on the physiological mechanisms of these two components.

Previous studies have demonstrated that HS cells in fact control the OMR^5^. Recently, Haikala *et al.* (2013)^3^ showed that optogenetic activation of HS cells located in one hemisphere of the brain elicited a head yaw response in *Drosophila*, suggesting that HS cell activity is sufficient to evoke this response. However, the precise quantitative relationship between changes in the membrane potential of HS cells and the OMR is poorly understood. In this study, we showed that membrane potential changes of HS cells in response to noisy motion stimuli showed proportional reductions with increasing noise intensity, that is, these responses did not resemble the noise-robust profile of both the discriminative performance revealed by ROC analysis and the head-turning amplitude of the OMR. One possible explanation for this discrepancy is that synaptic outputs of HS cells could have different dynamics than the membrane potential of cell bodies. It was recently reported that the change in HS cell membrane potential disagrees with the behavioral flight turning response, but the calcium accumulation in HS cell terminals is consistent with the behavioral response^3^. This study also proposed that this accumulation provides a mechanism for temporal integration of sensory input. These results also imply that the calcium level in terminals is not proportional to membrane potential changes. Further, a linear relationship between calcium levels in the terminals of VS cells and postsynaptic spike rates in V1 cells has been reported in the blowfly^4^. Presumably, non-linear and noise-robust neurotransmitter release depending on presynaptic calcium levels might linearly modulate the postsynaptic spike rate. Such molecular mechanisms might provide the robust OMR to noisy motion stimuli.

By applying ROC analysis, we were able to quantitatively compare neural activity with behavior and show robust discriminative performance to noisy stimuli with both behavior and HS cells activity. ROC analysis has been used to directly compare neural activities and psychophysical decisions in primates^9^. A correspondence between neural activities and behavioral or psychophysical judgments has been reported for a two-alternative forced choice test of stochastic motion stimuli in macaque monkeys^4^. These results are qualitatively similar to ours. Compared to primates, the precise neural network of the *Drosophila* visual system has been morphologically and functionally identified, which allowed applying physiological approaches such as whole-cell recording *in vivo.* Due to the relatively small number of neurons compared to primates and the abundance of helpful genetic tools, *Drosophila* provides an ideal model system to investigate the detailed neuronal mechanisms underlying the processing and representation of unreliable information and its transformation to appropriate psychophysical decisions. Further research on robust perception in both vision and other sensory modalities, such as olfaction and audition, would enhance our understanding of the neural mechanisms required to achieve robust information processing in the brain.

## Methods

### Flies

Fly stocks were reared on conventional medium that included cornmeal (Oriental Yeast Inc., Tokyo, Japan), yeast (Asahi Food and Health Care, Tokyo, Japan), and agar (Ina, Nagano, Japan) at 24 °C under a 12:12-h light/dark schedule. Female adult flies 1–4 days after eclosion were selected for use in all experiments. We used wild-type *Canton-S* for behavioral experiments and UAS-GCamp3 R27B03-Gal4 (a generous gift from Dr. Vivek Jayaraman) to label all HS cells for electrophysiological studies.

### Visual display and stimuli

We used an LED insect arena system^5^ (Metrix Technology Corp., New York, NY, USA). The system consists of a green LED display spanning 360° in azimuth 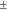
60° (96 × 16 pixels) for behavioral experiments and spanning 300° in azimuth and 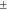
60° (80 × 16 pixels) for electrophysiological studies to present flies with horizontally moving vertical stripe patterns. These patterns were moved by turning the LED light on and off; therefore, they did not move continuously. Due to this manipulation, spike-like changes in membrane potential were often elicited^3^.

To build motion stimuli with noise, we superimposed a random dot pattern, which changed independently from frame to frame, on the vertically striped square-wave gratings that moved in a CW and CCW direction (stripe width; 8 pixels, approximately 30° per cycle). We modified the specific intensity of the random dots (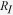: 0.2, 0.4, 0.6, 0.8, 1.0) to control the SNR of visual motion. In each frame, an LED is selected with a probability of 0.4 and turned on as a random dot. If this random dot LED is located on a bright bar in the original stripe pattern, the intensity of the random dot (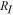) is subtracted from the bright bar intensity. Thus, although the total luminance fluctuated slightly between frames, the average luminance of the LED display was kept nearly constant during stimulus presentation. We defined the SNR for the motion stimulus as 
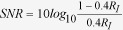
.

### Behavioral experiments

We measured head yaw angle as an indicator of the OMR as described previously^6^. Briefly, cold-anesthetized flies were tethered at the thorax to a steel pin with glue. In our experiments, the flies were unable to fly because their wings were fixed with glue. To minimize body movement without affecting head movement, the legs were stuck together with nail polish. The fly was placed in the center of the LED arena. Head movements were recorded with a CCD camera (Hamamatsu Photonics Inc., Hamamatsu, Japan). Images were collected at a frame rate of 25 or 30 Hz. After the video recordings, we used tracing software (PTV, Digimo, Japan) to measure the head yaw angle around the dorsoventral axis as the OMR. The stripe pattern turned for a duration of 10 s in each CW and CCW direction. The optimal frequency to elicit an OMR in *Drosophila* is around 4 Hz^6^. In this study, we used 1 and 4 Hz. The intervals between trials lasted a minimum of 5 s.

### Whole-cell patch clamp recordings

Preparation of flies and recording protocols were modified according to a previously published protocol^7^. Briefly, flies were cold-anesthetized and attached to a hole in the center of Parafilm sheet with the head bent down using two-component silicon glue (KWIK-SIL, WPI, Sarasota, FL, USA). To reduce the stress caused by the restraint, we did not clip all six legs or glue the proboscis to the head. However, the fly did not move during recording.

After attaching the fly to the holder, a portion of cuticle was removed in saline (130 mM NaCl, 5 mM KCl, 2 mM MgCl_2_[6H_2_O], 2 mM CaCl_2_[2H_2_O], 36 mM saccharose, and 5 mM HEPES [pH 7.3]). To remove the rest of the covering tissue, collagenase (0.5 mg/ml, Yakult, Tokyo, Japan) dissolved in extracellular saline was added locally just above the HS cell somata with a micropipette using positive pressure for approximately 30 s.

We performed whole-cell patch-clamp recordings in HS cells of the right brain hemisphere. The membrane potential was recorded by a patch-clamp amplifier (Axopatch 1D, Axon Instruments, Foster City, CA, USA). The recording electrode had a 7–12 M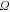 resistance, and the sampling frequency was 10 kHz. HS cells were observed under a microscope (BX51WI, Olympus) with a 40× water-immersion objective (RAMPlan FL N; Olympus, Tokyo, Japan). Patch-clamp electrodes contained an intracellular solution comprised of 140 mM K-aspartate, 10 mM HEPES, 1 mM KCl, 4 mM MgATP, 1 mM EGTA, and 0.5 mM NaGTP (pH 7.3). In the experiment shown in Fig. 3B, the intracellular solution included 6 mM biocytin-hydrazide to visualize the HS cell’s morphology. Because we did not include biocytin for the other experiments, we could not visually identify whether the recorded cell was one of the three HS cell types. However, no obvious differences were observed in the physiological responses during individual recordings.

The stripe pattern turned for 1 s in each CW (PD) or CCW (ND) direction at a temporal frequency of 1 Hz, which is near the optimal frequency for *Drosophila* tangential cells in quiescent animals^3^. A total of 12 motion patterns were presented in a pseudorandom order. The same stimulation pattern was given at least eight times for each cell.

### Immunohistochemistry

Figure 3 shows the visualization of a biocytin-filled neuron. After recordings, the brain was fixed with 4% paraformaldehyde for 30 min at 4 °C and washed three times with PBST (0.1 M phosphate-buffered saline containing 0.2% Triton X-100). It was blocked with 5% normal goat serum (NGS) for 1 h and then incubated with a primary antibody solution containing 1:30 mouse anti-nc82 (Hybridoma Bank, Iowa City, IA, USA) at 4 °C for 2 days. After washing with PBST, the brain was incubated with a secondary antibody solution containing 1:200 goat anti-mouse Alexa 633 and 1:500 streptavidin Alexa 555 (Molecular Probes, Eugene, OR, USA) at 4 °C for 2 days. After washing with PBST, it was mounted in 400 μl Vectashield medium (Vector Laboratories, Burlingame, CA, USA). Confocal images were acquired with an Olympus FV1000D IX81 confocal laser scanning microscope under 40 × magnification.

### Computer simulations

We constructed a 2-Quadrant-Detector model^8^ to reproduce HS activities in response to wide-field noisy motion stimuli. Reportedly, this model can semi-qualitatively reproduce a variety of experimental data measured from *Drosophila* tangential cells^9^.

To synthesize visual stimuli for the model, we needed to estimate how the image of the LED display is projected onto the fly retina. As shown in Fig. 4, the retinal image is calculated from the actual geometric arrangement of the LED display. In each frame of the synthesized visual stimuli, the luminance of each pixel is represented by a dimensionless integer value ranging from 0 to 5. The image of each frame is filtered by a 2D Gaussian function with a 7.5° standard deviation to mimic the receptive fields of lamina cells, which are formed by lateral inputs from different retina cells of six neighboring ommatidia^8^. After spatial filtering, the signal is passed through a sigmoid transfer function that represents typical non-linear properties including cell membrane potential responses, synaptic transmission, and habituation to stimuli in the fly’s visual system. We used the sigmoid transfer function defined as 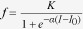, where 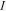 is the filtered signal, 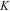 is a scaling factor, 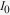 is a threshold, and 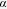 is a gain (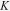 = 5, 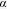 = 2, 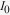 = 2.5).

The sigmoid output was processed by the 2-Quadrant-Detector model composed of a 2D motion detector array, which consisted of two subunits of horizontal and vertical local motion detectors located at lattice points in the 2D-array. Fig. 4a shows the architecture of each motion detector. HP is a temporal first-order high-pass filter (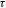 = 250 ms), and DC is a direct connection that passes 10% of the original signal. The sum of these is passed through two kinds of half-wave rectifiers that mimic the response properties of L1 and L2 cells^1^. The ON and OFF pathways correspond to the L1 and L2 cells in the lamina, respectively. Note that the clip point of the OFF pass rectifier was slightly shifted to the positive by 0.05 in reference to Eichner *et al.* (2011)^8^. Therefore, the OFF pathway signal involves a small amount of the ON signal. The outputs of the rectifiers are sent to the next processing stage composed of the standard Reichardt model (EMD), which consists of a first-order low pass-filter (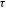 = 150 ms), a multiplication, and an imbalanced subtraction (positive:negative ratio of 1.0:0.8). The parameters of our model were estimated by manual fitting of the experimental results. At first, the parameters of the 2-Quadrant-Detector model without the two filters were estimated by fitting the model response to the average response of HS cells in zero noise conditions (i.e., presenting the stripe patterns). After that, while fixing the parameters of a part corresponding to the original 2-Quadrant-Detector model in the expanded model with the two filters, the parameters of the spatial filter and the sigmoid function were estimated in zero noise conditions. Compared to the parameters used in Eichner *et al.* (2011)^8^, the time constant of the low-pass filter was slightly larger, and the negative proportion in the imbalanced subtraction was slightly smaller. If we selected the parameters used in Eichner *et al.* (2011)^8^, the model’s residual activity after PD stimulation and ND stimulus response would not match our experimental results.

Tangential cells spatially integrate the output of local motion detectors on their dendrites and have receptive fields with a characteristic sensitivity distribution^3^. To keep the model relatively simple, the receptive field was approximated by the weighed summation of the horizontal and vertical local motion detector outputs. The membrane potential of the model, 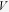, was determined by the following formula:
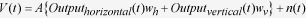
where 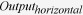 and 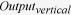 are the outputs of the horizontal and vertical local motion detectors, respectively; and 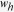 and 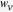 denote the weights of the horizontal and vertical components, respectively. 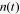 denotes a fluctuation of membrane potential described as follows. Fig. 4b shows the vector field of the weight vectors 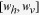 depending on the position, which were determined to reproduce the receptive field of the actual HS cell^4^. 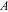 is a scaling factor, which was determined as 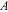 = 0.004.

To reproduce the fluctuation of membrane potential observed in HS cells, we added a colored noise to the model output. We generated the colored noise signal using a first-order autoregressive model expressed as: 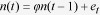, where 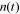 is the noise at time t, 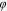 is a damping parameter determining the correlation length of the generated noise, and 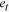 is a white noise with variance 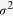. We recorded the spontaneous membrane activity of the HS cell for 5 s and estimated these parameters by fitting the spontaneous activity with the Yule-Walker method. The estimates of these are 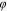 = 0.9974 and 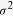 = 0.0022. Numerical simulations were carried out with MATLAB (MathWorks, Inc., Natick, MA, USA).

### Data analysis

To quantify the behavioral and neuronal discrimination performance between PD and ND motion stimuli, we obtained histograms of both head yaw rotation and HS cell membrane potential and calculated ROC curves for the PD and ND response histograms. In the behavioral experiments, the histograms in Fig. 2a to specific stimuli were calculated from data of the last 4 s during stimulus presentation for all examined flies. For electrophysiology, the histograms in Fig. 3e were calculated from data of all trials in each cell. Therefore, we obtained only one set of AUC from the behavioral experiments, while we got multiple AUCs from electrophysiology. To reduce the variance in each trial, we subtracted the baseline from the raw data in each trial for both the behavioral and electrophysiological experiments. We defined the behavioral baseline as the average head yaw angle during the total experimental time in each trial and defined the electrophysiological baseline as the average membrane potential during the 5 s before motion stimulus onset in each trial.

Next, we calculated the AUC. Because the AUC is mathematically equal to the probability for a correct answer in a two-alternative forced choice test, it is a good indicator for quantifying discrimination performance. When the ROC curve lies along the diagonal, the AUC is 0.5, suggesting that the fly cannot distinguish between two directions. When the ROC curve hugs the left axis and upper limit, the AUC approaches 1.0, indicating that the fly can fully distinguish between the two directions. These calculations were carried out with the Perfcurve function of MATLAB.

## Author Contributions

Y.S.Z., T.A., and T.K.M. conceived and designed the research. Y.S.Z. and H.I. carried out experiments and data analysis. Y.S.Z., T.M.Y., H.M., Y.S.K., T.A. and T.K.M. contributed reagents, materials, and analysis tools. Y.S.Z., T.A. and T.K.M. documented the findings.

## Additional Information

**How to cite this article**: Suzuki, Y. *et al.* Noise-robust recognition of wide-field motion direction and the underlying neural mechanisms in *Drosophila melanogaster*. *Sci. Rep.* **5**, 10253; doi: 10.1038/srep10253 (2015).

## Supplementary Material

Supplementary video S1

Supplementary video S2

Supplementary video S3

Supplementary video S4

Supplementary video S5

Supplementary video S6
